# Supplementary material for: Performance of molecular inversion probe DR23K and Paragon MAD4HatTeR Amplicon sequencing panels for detection of Plasmodium falciparum mutations associated with antimalarial drug resistance
Source: Malar J. 2025 Jun 12;24:188. doi: 10.1186/s12936-025-05441-3 (PMC12164144; doi:10.1186/s12936-025-05441-3)
Supplement: Supplementary file 1 — Additional file 1 [file 12936_2025_5441_MOESM1_ESM.pdf]

**Supplemental Table 1.** Molecular inversion probes included in the DR23K panel. Vol column indicates the volume (in μL) of 10 μM probe stock pooled for phosphorylation as described in **Supplemental Methods**.

| MIP | Vol | Targeted Region | Probe Sequence |
|---|---|---|---|
| PM2_S0_Sub0_mip0 | 4 | chr14:293316-293526 | AACATTTGATGGATTAAACATTGACAATTCNNNNAGATCGGAAGAGCACACGTGACTCGCCAAGCTGAAGNNNNNNNNNNNAGAGTAATCCATAATAATTTGTATGAGA |
| PM2_S0_Sub0_mip1 | 2 | chr14:293507-293714 | ATGTTTAAAATCATGTTCTCTTACTGTAATATCCANNNNAGATCGGAAGAGCACACGTGACTCGCCAAGCTGAAGNNNNNNNNNNNGTGCATTTTCAACTTTAAATCCAA |
| PM2_S0_Sub0_mip2 | 2 | chr14:293696-293906 | TTGGAGATAACCAACAACCATTTACANNNNAGATCGGAAGAGCACACGTGACTCGCCAAGCTGAAGNNNNNNNNNNNAAGAGATAATGAAATGAATGAAATTTTAAAA |
| PM2_S0_Sub0_mip3 | 2 | chr14:293861-294062 | GATATTATCATTTGAACTACCTAAATAATTTGTTTNNNNAGATCGGAAGAGCACACGTGACTCGCCAAGCTGAAGNNNNNNNNNNNCAGTTCCTGACACATAATTCATTT |
| PM2_S0_Sub0_mip4 | 2 | chr14:294045-294227 | AAGATTTATCAATAGGTTCAGTAGATCCAANNNNAGATCGGAAGAGCACACGTGACTCGCCAAGCTGAAGNNNNNNNNNNNGATTCATCTAAATCAAGAACATATGAAA |
| PM2_S0_Sub0_mip5 | 2 | chr14:294152-294357 | AATAAATTTATATGGAAGAGATAAATTACCAACAGNNNNAGATCGGAAGAGCACACGTGACTCGCCAAGCTGAAGNNNNNNNNNNNGGTCCTTCATAAAATCTTTCTTCAATA |
| PM2_S0_Sub0_mip6 | 2 | chr14:294316-294515 | ACTTTTTAAATAAAATGTTGCAGAATTTAGATGTNNNNAGATCGGAAGAGCACACGTGACTCGCCAAGCTGAAGNNNNNNNNNNNAAATGCTCTTTTCACCTTTTACTT |
| PM2_S0_Sub0_mip7 | 2 | chr14:294434-294640 | TAAAGTTATTTGCCAATATAAATCGTGGTTNNNNAGATCGGAAGAGCACACGTGACTCGCCAAGCTGAAGNNNNNNNNNNNAAGGTAGTATTCAGGTTCTAATGT |
| PM2_S0_Sub0_mip8 | 2 | chr14:294632-294843 | TGGAAACCAAAGGAAAAACATAATTATATAAGNNNNAGATCGGAAGAGCACACGTGACTCGCCAAGCTGAAGNNNNNNNNNNNCAACTTTTGAATTTACCTCAGAAAAT |
| PM2_S0_Sub0_mip9 | 2 | chr14:294700-294914 | ATACATAATCCTGGACCAACATCTTCNNNNAGATCGGAAGAGCACACGTGACTCGCCAAGCTGAAGNNNNNNNNNNNGGTTCATTATGGATAAAATATGTTTTCT |
| PM3_S0_Sub0_mip0 | 2 | chr14:297348-297544 | AACTAAAGTAAAAAGATGGAATGCTAAAAGNNNNAGATCGGAAGAGCACACGTGACTCGCCAAGCTGAAGNNNNNNNNNNNTTGAAAGCCGTAAAAATAAAAGTTAAA |
| PM3_S0_Sub0_mip1 | 2 | chr14:297501-297680 | GGTAAAATCTTCTTCTTTAATGGTTAAATTCANNNNAGATCGGAAGAGCACACGTGACTCGCCAAGCTGAAGNNNNNNNNNNNCTGTCGAATATTTTGAAGTCTTAATTAT |
| PM3_S0_Sub0_mip10 | 4 | chr14:298754-298965 | TATATATTTGTGATGTCGTTTGATTATATTGCNNNNAGATCGGAAGAGCACACGTGACTCGCCAAGCTGAAGNNNNNNNNNNNTTTGTTCTAGGTGACCCATTTAT |
| PM3_S0_Sub0_mip2 | 2 | chr14:297615-297826 | TTATTTAGGTAGTGAGTTTGATAATGTGGANNNNAGATCGGAAGAGCACACGTGACTCGCCAAGCTGAAGNNNNNNNNNNNATTATTTGTGACAGTTTTCATAGTTTTG |
| PM3_S0_Sub0_mip3 | 2 | chr14:297773-297967 | TTTAATTTATGCTCTTTAATTGTTTTCATAAGTCTNNNNAGATCGGAAGAGCACACGTGACTCGCCAAGCTGAAGNNNNNNNNNNNTTCACAAGACTCTGATGTACATTTTATA |
| PM3_S0_Sub0_mip4 | 2 | chr14:297891-298076 | ACAATAAGTGGAATATTTAGTAAAGATTTAGTAACNNNNAGATCGGAAGAGCACACGTGACTCGCCAAGCTGAAGNNNNNNNNNNNAGATTTAGCAAATGTATTATCTTTTGGA |
| PM3_S0_Sub0_mip5 | 2 | chr14:298048-298240 | TATCATCTTTTTCATATGTTTTTGATTTTGATGAANNNNAGATCGGAAGAGCACACGTGACTCGCCAAGCTGAAGNNNNNNNNNNNGTGTTTTTAATTCTACAATATATGGATCTATA |
| PM3_S0_Sub0_mip6 | 2 | chr14:298153-298359 | AAAGATTCTTTGATGGACCATTGAACNNNNAGATCGGAAGAGCACACGTGACTCGCCAAGCTGAAGNNNNNNNNNNNTCAGTACCCTATAAATTTATTGAAATGA |
| PM3_S0_Sub0_mip7 | 2 | chr14:298339-298537 | AATAACCTTTATTTTTGTTTTCTGGTGGTNNNNAGATCGGAAGAGCACACGTGACTCGCCAAGCTGAAGNNNNNNNNNNNAAGAATGGAACTTTGAAAACACTT |
| PM3_S0_Sub0_mip8 | 2 | chr14:298434-298629 | TATATACTTTAGAACCTAAACAATACCTTGAANNNNAGATCGGAAGAGCACACGTGACTCGCCAAGCTGAAGNNNNNNNNNNNATGTGGCAAGTTGATTTAGATGTA |
| PM3_S0_Sub0_mip9 | 2 | chr14:298589-298798 | TTACCACAAGTGGTTACATACAAAGANNNNAGATCGGAAGAGCACACGTGACTCGCCAAGCTGAAGNNNNNNNNNNNGCTTTATAAATTTTTGGCTAAAGCAAAT |
| crt_S0_Sub0_mip1 | 4 | chr7:403099-403307 | ATTTTAAAATATTTATGTCATATATGTGGAAGACANNNNAGATCGGAAGAGCACACGTGACTCGCCAAGCTGAAGNNNNNNNNNNNTCCATTTTTGGATACTTACTTCCT |
| crt_S0_Sub0_mip10 | 4 | chr7:404323-404479 | AACAATAATAACTGCTCCGAGATAATTGTNNNNAGATCGGAAGAGCACACGTGACTCGCCAAGCTGAAGNNNNNNNNNNNATAATAAAAACAAAGTTTAAGTGTTAATATATAT |
| crt_S0_Sub0_mip11 | 4 | chr7:404375-404577 | TTTCTTCTTGTGTTTCAAAAGATAATTTCANNNNAGATCGGAAGAGCACACGTGACTCGCCAAGCTGAAGNNNNNNNNNNNTATTTCCCTTGTCATGTTTGAAAA |
| crt_S0_Sub0_mip12 | 2 | chr7:404588-404802 | TGTTTGAAAAGCATACAGGCTAAAAANNNNAGATCGGAAGAGCACACGTGACTCGCCAAGCTGAAGNNNNNNNNNNNTGGAAGGGTGTATACAGGTAATAT |
| crt_S0_Sub0_mip13 | 2 | chr7:404756-404915 | GTATAATTTTCCCTTTTTAGTTCATTTACCATNNNNAGATCGGAAGAGCACACGTGACTCGCCAAGCTGAAGNNNNNNNNNNNAACGCATTATAATTATTTCTGTTATTTTTATT |
| crt_S0_Sub0_mip14 | 4 | chr7:404809-404999 | TAATATAAGACAAGAAGTGAACAATTGGAANNNNAGATCGGAAGAGCACACGTGACTCGCCAAGCTGAAGNNNNNNNNNNNTACCTCTACGACTGTGTTTCTT |
| crt_S0_Sub0_mip15 | 2 | chr7:404997-405192 | ATGGGTAAGAAGCTTATAATAAAATTTCAAAANNNNAGATCGGAAGAGCACACGTGACTCGCCAAGCTGAAGNNNNNNNNNNNAAATGGTTTCGCATGTTTATTCTT |
| crt_S0_Sub0_mip16 | 2 | chr7:405158-405363 | AAGACCACAATTCTGAAGAGGAAACANNNNAGATCGGAAGAGCACACGTGACTCGCCAAGCTGAAGNNNNNNNNNNNCATAGCTGGTTATTAAATTATCACAAAT |
| crt_S0_Sub0_mip17 | 2 | chr7:405353-405571 | ATTGTTAGTTGTATACAAGGTCCAGCNNNNAGATCGGAAGAGCACACGTGACTCGCCAAGCTGAAGNNNNNNNNNNNTTAGAAAACCTTCGCATTGTTTT |

| | | | |
|---|---|---|---|
| crt_S0_Sub0_mip18 | 2 | chr7:405569-405736 | ATATGTCATGGTAGAAAATTTGTCGATAATNNNNAGATCGGAAGAGCACACGTGACTCGCCAAGCTGAAGNNNNNNNNNNTACGTTGTACCATCATAAACATTTAAAT |
| crt_S0_Sub0_mip19 | 2 | chr7:405653-405843 | CAAGATTATTAGATTTCGTAACTTTGGTAAGNNNNAGATCGGAAGAGCACACGTGACTCGCCAAGCTGAAGNNNNNNNNNNCTTAGCCGTAAGAATTAAAAAGATATAA |
| crt_S0_Sub0_mip2 | 4 | chr7:403184-403345 | AATGAATGTATAATAAATAATATGACTGCGTGNNNNAGATCGGAAGAGCACACGTGACTCGCCAAGCTGAAGNNNNNNNNNNCCAATAGGTTGATTTATCTATTCATTTA |
| crt_S0_Sub0_mip20 | 4 | chr7:405837-406030 | TACAACATCACCCTATAAAATTAAAAAGTAAAAATNNNNAGATCGGAAGAGCACACGTGACTCGCCAAGCTGAAGNNNNNNNNNNCCTACACGGTAAATTATAGAACCA |
| crt_S0_Sub0_mip21 | 4 | chr7:405979-406163 | ATTTTTATATTTCCATCTGTCTTTTTATTCTATTGNNNNAGATCGGAAGAGCACACGTGACTCGCCAAGCTGAAGNNNNNNNNNNATGGCTTGTTCGTTCATAAATATTTA |
| crt_S0_Sub0_mip22 | 2 | chr7:406082-406280 | TTTGTATTACTTTCTAAGATAATATTTCCTACACGNNNNAGATCGGAAGAGCACACGTGACTCGCCAAGCTGAAGNNNNNNNNNNTTGAATCGACGTTGGTTAATTCT |
| crt_S0_Sub0_mip23 | 4 | chr7:406274-406465 | AATAATGTTGAAGGTATAGAGATCTCTTTATTNNNNAGATCGGAAGAGCACACGTGACTCGCCAAGCTGAAGNNNNNNNNNNAAATGAGAAATGAAGAAAATGAAGATT |
| crt_S0_Sub0_mip3 | 4 | chr7:403291-403507 | GAGGTTCTTGTCTTGGTAAATGTGCNNNNAGATCGGAAGAGCACACGTGACTCGCCAAGCTGAAGNNNNNNNNNNAATGACGAGCGTTATAGAGAATTA |
| crt_S0_Sub0_mip4 | 2 | chr7:403492-403702 | ATCTGTTAAGGTCGACAAGGGAAANNNNAGATCGGAAGAGCACACGTGACTCGCCAAGCTGAAGNNNNNNNNNNAAGGAATAAACAATAAAGAACATAATCA |
| crt_S0_Sub0_mip5 | 2 | chr7:403592-403787 | GATTTATAAGAGAATCTATTCCACCTACCANNNNAGATCGGAAGAGCACACGTGACTCGCCAAGCTGAAGNNNNNNNNNNAGAGATTAAGGATAATATTTTTATTTATATTTTAA |
| crt_S0_Sub0_mip6 | 2 | chr7:403780-403972 | TAATATATTGATTTATCTTACTTTTGAATTTCCCTNNNNAGATCGGAAGAGCACACGTGACTCGCCAAGCTGAAGNNNNNNNNNNGAACAGGCATCTAACATGGATATA |
| crt_S0_Sub0_mip7 | 2 | chr7:403933-404082 | TCTTCTGCTTTTTAATATTAAGATATAGGTAAGTANNNNAGATCGGAAGAGCACACGTGACTCGCCAAGCTGAAGNNNNNNNNNNTTGTTTATATATTTATATTTATTTCTTATGACCTT |
| crt_S0_Sub0_mip8 | 4 | chr7:404081-404279 | ATATTAATAGGAATACTTAATTGAAGAACAAATGANNNNAGATCGGAAGAGCACACGTGACTCGCCAAGCTGAAGNNNNNNNNNNTCCGAGATAATTGTATAAGTGATATCTA |
| crt_S0_Sub0_mip9 | 4 | chr7:404129-404324 | AACAATAGCTCTTGTAGAAATGAAATTATCTTNNNNAGATCGGAAGAGCACACGTGACTCGCCAAGCTGAAGNNNNNNNNNNTTAATATTAAGATATAGGTAAGTATACTATTTTAA |
| dhfr-ts_S0_Sub0_mip1 | 2 | chr4:748093-748266 | AGTTACAACATATGTGAATGAATCAAAATATGNNNNAGATCGGAAGAGCACACGTGACTCGCCAAGCTGAAGNNNNNNNNNNTTATTTATATATTTATATTTCTCCTTTTTATGAT |
| dhfr-ts_S0_Sub0_mip10 | 2 | chr4:749309-749518 | AATGTAAAAGATCTTGACCAAATGGCNNNNAGATCGGAAGAGCACACGTGACTCGCCAAGCTGAAGNNNNNNNNNNAATGTAAGGATATGGGAAGCTAAT |
| dhfr-ts_S0_Sub0_mip11 | 2 | chr4:749460-749638 | TTTTTAATTGATCCACTCCTTTATTTTCATAATNNNNAGATCGGAAGAGCACACGTGACTCGCCAAGCTGAAGNNNNNNNNNNTAGAATAAGAAGCAATATTAAAAGGTAC |
| dhfr-ts_S0_Sub0_mip12 | 2 | chr4:749579-749756 | TTGATAGTTTAAAAATTCAACTTAACAGAATACCCNNNNAGATCGGAAGAGCACACGTGACTCGCCAAGCTGAAGNNNNNNNNNNCCTTGTCATATTTTATGTCAGTTTTAT |
| dhfr-ts_S0_Sub0_mip13 | 2 | chr4:749711-749926 | CGCAGGTTGCAAATTACAGACTTGTNNNNAGATCGGAAGAGCACACGTGACTCGCCAAGCTGAAGNNNNNNNNNNATTTTGTCATCATTTGTTCATATATTTTT |
| dhfr-ts_S0_Sub0_mip2 | 2 | chr4:748114-748299 | AAAACGTCGCAGACTTGTTCCANNNNAGATCGGAAGAGCACACGTGACTCGCCAAGCTGAAGNNNNNNNNNNGTTTAAATATTTACATCTCTTATATTTCAATTTT |
| dhfr-ts_S0_Sub0_mip3 | 2 | chr4:748217-748428 | TTTATTTCCTAGACCTCTAAATGTGTAGTTNNNNAGATCGGAAGAGCACACGTGACTCGCCAAGCTGAAGNNNNNNNNNNATCCTATTGCTTAAAGGTTTAAATTTT |
| dhfr-ts_S0_Sub0_mip4 | 2 | chr4:748374-748585 | TCCGTTGTTTATCAAGAATTTTTAGAAAAGNNNNAGATCGGAAGAGCACACGTGACTCGCCAAGCTGAAGNNNNNNNNNNGATAATGTAAATGATATGCCTAATTCTA |
| dhfr-ts_S0_Sub0_mip5 | 2 | chr4:748548-748746 | CAAGTAAAACTATTAGATCTTCAACTTTGTTNNNNAGATCGGAAGAGCACACGTGACTCGCCAAGCTGAAGNNNNNNNNNNCGTTTTCTTATAAATGATAAAATCCAAT |
| dhfr-ts_S0_Sub0_mip6 | 2 | chr4:748719-748908 | TAAAATTAATTATGAAAATGATGATGATGATGAAGNNNNAGATCGGAAGAGCACACGTGACTCGCCAAGCTGAAGNNNNNNNNNNTGAAAATGAGTATCAAATTATTTCTGTTA |
| dhfr-ts_S0_Sub0_mip7 | 2 | chr4:748870-749072 | ATATGACATGTATCTTTGTCATCATTCTTTNNNNAGATCGGAAGAGCACACGTGACTCGCCAAGCTGAAGNNNNNNNNNNTTCCATTCATCATAATATCATAAATAATATTTA |
| dhfr-ts_S0_Sub0_mip8 | 2 | chr4:749038-749237 | TTTGGTTTATTAGAGGAGAAACAAATGGTNNNNAGATCGGAAGAGCACACGTGACTCGCCAAGCTGAAGNNNNNNNNNNCTATACATCCAAATGATTTTCAAATATATAATAG |
| dhfr-ts_S0_Sub0_mip9 | 2 | chr4:749183-749371 | ATATTGACTTAAATCAAATTTCATAATATATCCGANNNNAGATCGGAAGAGCACACGTGACTCGCCAAGCTGAAGNNNNNNNNNNATGTCTCCATTGAAAACCATAAAT |
| dhps_S0_Sub0_mip0 | 2 | chr8:548152-548314 | ACCTTGTCGAAAAATATTTAGGTAAGAAANNNNAGATCGGAAGAGCACACGTGACTCGCCAAGCTGAAGNNNNNNNNNNATAATAAATTTTGCGTAAAGAAATCTAAT |
| dhps_S0_Sub0_mip1 | 4 | chr8:548309-548472 | CAGTTTCTAGAATCAACACAGCGTNNNNAGATCGGAAGAGCACACGTGACTCGCCAAGCTGAAGNNNNNNNNNNGTGAAAAATAAAAATATATAGAATCAAAGAGT |
| dhps_S0_Sub0_mip10 | 2 | chr8:549625-549827 | AACTATAATGTTTTTAAAGAATGTGTTGATAATGNNNNAGATCGGAAGAGCACACGTGACTCGCCAAGCTGAAGNNNNNNNNNNAACCTAAACGTGCTGTTCAAA |
| dhps_S0_Sub0_mip11 | 2 | chr8:549823-549996 | TATCAATACTTATAATTGGTTTCGCATCACNNNNAGATCGGAAGAGCACACGTGACTCGCCAAGCTGAAGNNNNNNNNNNTTATATCATAAACTAGATTATCATAATTTGTTA |
| dhps_S0_Sub0_mip12 | 2 | chr8:549970-550149 | AAAATATACATGTATATGATGAGTATCCACTTNNNNAGATCGGAAGAGCACACGTGACTCGCCAAGCTGAAGNNNNNNNNNNTTCTATAGTGTAGTTCTAATGCATAAAA |

| | | | |
|---|---|---|---|
| dhps_S0_Sub0_mip13 | 2 | chr8:550078-550241 | GTATTCCATTTAATACAAGAAAATTTAATCTTTGTNNNNAGATCGGAAGAGCACACGTGACTCGCCAAGCTGAAGNNNNNNNNNNCATCATGTAATTTTTGTTGTGTATTTAT |
| dhps_S0_Sub0_mip14 | 4 | chr8:550203-550406 | AAATGAGTATACAAAAGTAACAATTCTATATATGTNNNNAGATCGGAAGAGCACACGTGACTCGCCAAGCTGAAGNNNNNNNNNNCCACTTTTTATTGGATATTCAAGAAAA |
| dhps_S0_Sub0_mip15 | 2 | chr8:550323-550526 | AAACATCCAATTGTGTGATTTGTCCNNNNAGATCGGAAGAGCACACGTGACTCGCCAAGCTGAAGNNNNNNNNNNGAACTCTTATTAGATCTACCTTTTTATAATA |
| dhps_S0_Sub0_mip16 | 4 | chr8:550515-550726 | ATTTGTTGTTCTTTTTCCTATTTTACATGCNNNNAGATCGGAAGAGCACACGTGACTCGCCAAGCTGAAGNNNNNNNNNNTTCTTTTCAGGTGGATTAGCAATT |
| dhps_S0_Sub0_mip17 | 4 | chr8:550580-550787 | GATTTTGTTTCTAAAACGTCATGAACTCNNNNAGATCGGAAGAGCACACGTGACTCGCCAAGCTGAAGNNNNNNNNNNAGTCCACTTTTGATAATTTTATAAAATCA |
| dhps_S0_Sub0_mip2 | 2 | chr8:548460-548609 | ATATATGATGTTAATTATATAAACGAATTGATGCANNNNAGATCGGAAGAGCACACGTGACTCGCCAAGCTGAAGNNNNNNNNNNCATACTTTTATATACATACCTTTATATACATACTT |
| dhps_S0_Sub0_mip3 | 2 | chr8:548575-548752 | TTTTATCTAATACAATGTATTCTGGAACGGNNNNAGATCGGAAGAGCACACGTGACTCGCCAAGCTGAAGNNNNNNNNNNTACATTCTAATAAATAATTTTCTACATTTACT |
| dhps_S0_Sub0_mip4 | 2 | chr8:548751-548955 | AAAATGTAAAAGAGAAAGAAAAATTTGAAAATCGTNNNNAGATCGGAAGAGCACACGTGACTCGCCAAGCTGAAGNNNNNNNNNNTGGAAAAGTTGATAATAGTATTCTAAAG |
| dhps_S0_Sub0_mip5 | 2 | chr8:548937-549125 | AATATATTTTATAACTACCAACATACTAAGAGGATNNNNAGATCGGAAGAGCACACGTGACTCGCCAAGCTGAAGNNNNNNNNNNATCTTTATCCATATTTACTTTAGACATATT |
| dhps_S0_Sub0_mip6 | 2 | chr8:549105-549309 | ATATTGAATTTTTATCCATTCCTCATGTGTNNNNAGATCGGAAGAGCACACGTGACTCGCCAAGCTGAAGNNNNNNNNNNACTCTCAAAATATATTCATTTGGAAAGA |
| dhps_S0_Sub0_mip7 | 2 | chr8:549299-549451 | TTGTTTATTATTTCTTGTGGATCTCTAAAAGANNNNAGATCGGAAGAGCACACGTGACTCGCCAAGCTGAAGNNNNNNNNNNTTTCTTTAATATTTATATTATATTGTTCTTTCATC |
| dhps_S0_Sub0_mip8 | 2 | chr8:549370-549583 | AGATGGAGGTATTTTTGTTGAACCTANNNNAGATCGGAAGAGCACACGTGACTCGCCAAGCTGAAGNNNNNNNNNNCAACACACAGATATAGCATACTTTTAT |
| dhps_S0_Sub0_mip9 | 2 | chr8:549583-549740 | AAAAAGAATCATAATTAACATTTAATATTCCAACANNNNAGATCGGAAGAGCACACGTGACTCGCCAAGCTGAAGNNNNNNNNNNCATTCCATTCTTTTTGAAATAATTGTAA |
| k13_S0_Sub0_mip1 | 3 | chr13:1724818-1724999 | TACACCATTTAGAAATTGCCATCTTTTATNNNNAGATCGGAAGAGCACACGTGACTCGCCAAGCTGAAGNNNNNNNNNNAAAAATAAGAACATTTAAAATTTCTTCATTT |
| k13_S0_Sub0_mip10 | 3 | chr13:1726199-1726378 | ATATTGAAGAACAGAAATTACATGATGAAAGANNNNAGATCGGAAGAGCACACGTGACTCGCCAAGCTGAAGNNNNNNNNNNATGATACTTATGAAAAGAAAATTATTGAAA |
| k13_S0_Sub0_mip11 | 3 | chr13:1726304-1726503 | AGACACAAATGAATTTTATTCGAGAAAAAGNNNNAGATCGGAAGAGCACACGTGACTCGCCAAGCTGAAGNNNNNNNNNNATATGAATTCTCCATCAATTATGAATAC |
| k13_S0_Sub0_mip12 | 5 | chr13:1726474-1726678 | ACATATTATTAAATGTTCTTGATAAATTACTTGGTNNNNAGATCGGAAGAGCACACGTGACTCGCCAAGCTGAAGNNNNNNNNNNTTAATCCAGAATCATCATTTATAAGATTT |
| k13_S0_Sub0_mip13 | 3 | chr13:1726531-1726741 | TATGAATTCTCCATCAATTATGAATACCAANNNNAGATCGGAAGAGCACACGTGACTCGCCAAGCTGAAGNNNNNNNNNNGAATCCATTGATATGAGTGTATTAGATT |
| k13_S0_Sub0_mip14 | 3 | chr13:1726693-1726878 | TTCTCGCTACTACTTCCGCTTTNNNNAGATCGGAAGAGCACACGTGACTCGCCAAGCTGAAGNNNNNNNNNNTTTCCTATATTATCTTTAGACATATTATTAAAT |
| k13_S0_Sub0_mip15 | 3 | chr13:1726824-1727000 | AATAGTTTCCTTTTAAATAATAGTAGTTATGGAAANNNNAGATCGGAAGAGCACACGTGACTCGCCAAGCTGAAGNNNNNNNNNNCATTCATTTATTATGTTTTTGTTAATTAAATTATA |
| k13_S0_Sub0_mip2 | 3 | chr13:1724927-1725137 | AAGTTCTAAATTCATGTCATTTCTTTTCACNNNNAGATCGGAAGAGCACACGTGACTCGCCAAGCTGAAGNNNNNNNNNNACAATTTCCATATGCCTTATTAGAA |
| k13_S0_Sub0_mip3 | 3 | chr13:1725079-1725274 | TAGGGGTATTCAAAGGTGCCACCNNNNAGATCGGAAGAGCACACGTGACTCGCCAAGCTGAAGNNNNNNNNNNGGAATCTAATATGTTATGTTCATTATCA |
| k13_S0_Sub0_mip4 | 3 | chr13:1725247-1725419 | TAATAAAATTTATGTCATTGGTGGAACTAATGGTNNNNAGATCGGAAGAGCACACGTGACTCGCCAAGCTGAAGNNNNNNNNNNGATGTATGGTATGTTTCAAGTAATTTAAATATA |
| k13_S0_Sub0_mip5 | 3 | chr13:1725363-1725575 | TGTACACATACGCCAGCATTGTTGNNNNAGATCGGAAGAGCACACGTGACTCGCCAAGCTGAAGNNNNNNNNNNCGGTATAATAGAAGAGCCATCATA |
| k13_S0_Sub0_mip6 | 3 | chr13:1725563-1725749 | AAAAAGCTTATTTTGGAAGTGCTGTATTGNNNNAGATCGGAAGAGCACACGTGACTCGCCAAGCTGAAGNNNNNNNNNNTAACTTCTTAAGAAATCCGTTAACTATA |
| k13_S0_Sub0_mip7 | 3 | chr13:1725714-1725926 | ACCAACATTAATATCAATCATAGTTTCAGTNNNNAGATCGGAAGAGCACACGTGACTCGCCAAGCTGAAGNNNNNNNNNNACCATAAAATTCTGCTTCTTTCAA |
| k13_S0_Sub0_mip8 | 3 | chr13:1725921-1726121 | ATTTTTGAAACATCTAGACATACCTTAACANNNNAGATCGGAAGAGCACACGTGACTCGCCAAGCTGAAGNNNNNNNNNNATTGATATATCTAATGGTTATAAACAAATAAAA |
| k13_S0_Sub0_mip9 | 3 | chr13:1726086-1726264 | ATAATTTATCTTTTTCTCGAATAAAATTCATTTGTNNNNAGATCGGAAGAGCACACGTGACTCGCCAAGCTGAAGNNNNNNNNNNCGATTTCTTGTAAAAATCTTAATCTTTC |
| mdr1_S0_Sub0_mip0 | 2 | chr5:957787-957958 | AACAAAAAGAGTACCGCTGAATTATTTAGANNNNAGATCGGAAGAGCACACGTGACTCGCCAAGCTGAAGNNNNNNNNNNGTGTACATAGCTTATTTCATTTATAAGATTT |
| mdr1_S0_Sub0_mip1 | 2 | chr5:957899-958080 | TTTACCCATCTTTCAACACAAAATCAAATNNNNAGATCGGAAGAGCACACGTGACTCGCCAAGCTGAAGNNNNNNNNNNGGTAATGTTCCTCCTGATAATACA |
| mdr1_S0_Sub0_mip10 | 2 | chr5:959125-959327 | ACAAATGCATATGTTTTCCCTTCTTTTANNNNAGATCGGAAGAGCACACGTGACTCGCCAAGCTGAAGNNNNNNNNNNTCCATTGCTTCTAAATCTTTTAAACTAT |
| mdr1_S0_Sub0_mip11 | 2 | chr5:959261-959476 | TCTGATGTTGTTGATGTGTCCAAAANNNNAGATCGGAAGAGCACACGTGACTCGCCAAGCTGAAGNNNNNNNNNNTTGAAATGGTGGAGATCAAAAATT |

| | | | |
|---|---|---|---|
| mdr1_S0_Sub0_mip13 | 2 | chr5:959453-959625 | AAATCCTAAAATTCTAATTCTTGATGAAGCTACNNNNAGATCGGAAGAGCACACGTGACTCGCCAAGCTGAAGNNNNNNNNNNNNTGACATCAAATGAATTATTAGAAATGAAA |
| mdr1_S0_Sub0_mip14 | 2 | chr5:959559-959748 | CTACTAAGGTATCATATTTATCTGGTAATGATGANNNNAGATCGGAAGAGCACACGTGACTCGCCAAGCTGAAGNNNNNNNNNNNNGGCATATCTTATAGTACTTAATCTATGT |
| mdr1_S0_Sub0_mip15 | 2 | chr5:959738-959909 | GTACACATGATAGTCTTATGAAAAATAAAAATGGTNNNNAGATCGGAAGAGCACACGTGACTCGCCAAGCTGAAGNNNNNNNNNNNNTTTGAAAGGAAATGAAAATAGAATAACT |
| mdr1_S0_Sub0_mip16 | 2 | chr5:959913-960073 | TACCTTGTTCAATAATATAGCTACCCTCANNNNAGATCGGAAGAGCACACGTGACTCGCCAAGCTGAAGNNNNNNNNNNNNTTCATTTTCATGTATTGATAAACTATTCAT |
| mdr1_S0_Sub0_mip17 | 2 | chr5:960000-960158 | AAGTTCCATTTTTCAAAAGAATGTTTAGAAGANNNNAGATCGGAAGAGCACACGTGACTCGCCAAGCTGAAGNNNNNNNNNNNNCAAAAGATTTCATCAAATAAATCTTCAAATA |
| mdr1_S0_Sub0_mip18 | 2 | chr5:960150-960344 | TTTCATTTTCTGCTGTATTTTTACAATTACGANNNNAGATCGGAAGAGCACACGTGACTCGCCAAGCTGAAGNNNNNNNNNNNNATTTGAGTTATATTCTAGATTTGCAAAAT |
| mdr1_S0_Sub0_mip19 | 2 | chr5:960315-960527 | ATCAAGATAAAAATACCCCAGGTGTTNNNNAGATCGGAAGAGCACACGTGACTCGCCAAGCTGAAGNNNNNNNNNNNNGGATTATATCCCGTATTTGCTTTAT |
| mdr1_S0_Sub0_mip2 | 2 | chr5:958064-958221 | AAATAATAATTTTCTATGTTGTGCAGGTAAACATNNNNAGATCGGAAGAGCACACGTGACTCGCCAAGCTGAAGNNNNNNNNNNNNTGTAATTACATCCATACAATAACTTGAT |
| mdr1_S0_Sub0_mip20 | 2 | chr5:960501-960664 | TATTTTCAAATAATCTACGTTTCATAGTCTTTTCGNNNNAGATCGGAAGAGCACACGTGACTCGCCAAGCTGAAGNNNNNNNNNNNNTGCAACAATTGGACAAAAATAAAA |
| mdr1_S0_Sub0_mip21 | 2 | chr5:960643-960807 | TTCAGATGATGAAATGTTTAAAGATCCAAGNNNNAGATCGGAAGAGCACACGTGACTCGCCAAGCTGAAGNNNNNNNNNNNNTTATTTTCTCTCATTTCATAATGCTCTT |
| mdr1_S0_Sub0_mip22 | 2 | chr5:960741-960916 | CTCTTACAGCAAATACACGCATATTAATNNNNAGATCGGAAGAGCACACGTGACTCGCCAAGCTGAAGNNNNNNNNNNNNGTCCTTTATTTTTATAATCAATAGCTTTTTC |
| mdr1_S0_Sub0_mip23 | 2 | chr5:960896-961052 | GAGGTACTATATTAGTTGATGACTTTATGAAATCCNNNNAGATCGGAAGAGCACACGTGACTCGCCAAGCTGAAGNNNNNNNNNNNNGCATACTGTTATTAATTATGGTTTAGAA |
| mdr1_S0_Sub0_mip24 | 2 | chr5:960984-961147 | CCCATAAAGCTGCATTTACAATAATTCTTNNNNAGATCGGAAGAGCACACGTGACTCGCCAAGCTGAAGNNNNNNNNNNNNCTCAAATGATAATTTTGCATTTTCTGA |
| mdr1_S0_Sub0_mip25 | 2 | chr5:961131-961350 | AAAAACTACAGCAATCGTTGGAGAAANNNNAGATCGGAAGAGCACACGTGACTCGCCAAGCTGAAGNNNNNNNNNNNNCTGGTAGTTATGCTGGAAAATTAA |
| mdr1_S0_Sub0_mip26 | 2 | chr5:961292-961452 | ACGGAAATTTACATCTTTAATATCAACTTTACCTNNNNAGATCGGAAGAGCACACGTGACTCGCCAAGCTGAAGNNNNNNNNNNNNTGATAATCTTGAAAATTTGTCATATCATTT |
| mdr1_S0_Sub0_mip27 | 2 | chr5:961444-961653 | TCAAGAACCCATGTTATTTAATATGTCCANNNNAGATCGGAAGAGCACACGTGACTCGCCAAGCTGAAGNNNNNNNNNNNNGATTTTATGACTTGAAAAATGATCACAT |
| mdr1_S0_Sub0_mip28 | 2 | chr5:961545-961755 | ATCCAGATTGGTTTGAAAATTCATTTACATNNNNAGATCGGAAGAGCACACGTGACTCGCCAAGCTGAAGNNNNNNNNNNNNTGGTAATGATTCGATAAATTCATCTATA |
| mdr1_S0_Sub0_mip29 | 2 | chr5:961715-961882 | ATATTATTATTAGATGAAGCAACATCATCACTTGANNNNAGATCGGAAGAGCACACGTGACTCGCCAAGCTGAAGNNNNNNNNNNNNGAAAATATCAAATTTGGAAGAGAAGAT |
| mdr1_S0_Sub0_mip3 | 4 | chr5:958145-958352 | CATGTTCTTTAATATTACACCAAACACAGANNNNAGATCGGAAGAGCACACGTGACTCGCCAAGCTGAAGNNNNNNNNNNNNGAACTCACTTGTTCTAAATAAAAATCTA |
| mdr1_S0_Sub0_mip30 | 2 | chr5:961817-962026 | ACCATATGGTCCAACATTTGTATCATNNNNAGATCGGAAGAGCACACGTGACTCGCCAAGCTGAAGNNNNNNNNNNNNTCAGGGTTATTAAATACCACAATTTT |
| mdr1_S0_Sub0_mip31 | 3 | chr5:962018-962199 | TTTATGCATTTATATGATCCGCAAACATNNNNAGATCGGAAGAGCACACGTGACTCGCCAAGCTGAAGNNNNNNNNNNNNGCCCACAGAATTGCATCTATAAAA |
| mdr1_S0_Sub0_mip4 | 2 | chr5:958331-958498 | AGGATTATTATCATGAAATTGTCCATCTTGNNNNAGATCGGAAGAGCACACGTGACTCGCCAAGCTGAAGNNNNNNNNNNNNACATATGACACCACAAACATAAATTAA |
| mdr1_S0_Sub0_mip5 | 2 | chr5:958409-958619 | TTGCAAGTTATTGTGGAGAAAAGACTNNNNAGATCGGAAGAGCACACGTGACTCGCCAAGCTGAAGNNNNNNNNNNNNAGGAATTGGTACGAAATTTATAACAATT |
| mdr1_S0_Sub0_mip6 | 2 | chr5:958606-958819 | ATTAAAGCCTCTTCTATAATGGACATGGTNNNNAGATCGGAAGAGCACACGTGACTCGCCAAGCTGAAGNNNNNNNNNNNNTGAGGCACCATTAAAATCATTATT |
| mdr1_S0_Sub0_mip7 | 2 | chr5:958773-958971 | ATTAGTTGAAAATAATGATGATGGAGAAACATTACNNNNAGATCGGAAGAGCACACGTGACTCGCCAAGCTGAAGNNNNNNNNNNNNTAGTTTCTTATGCATTCGGTTTTT |
| mdr1_S0_Sub0_mip8 | 2 | chr5:958940-959143 | TGTTGCTTCTAAAGCTTTCATATATTCTGTTATATNNNNAGATCGGAAGAGCACACGTGACTCGCCAAGCTGAAGNNNNNNNNNNNNTCTTTCAATTAATTTTAGTATGGTTGATTT |
| mdr1_S0_Sub0_mip9 | 2 | chr5:959072-959253 | AAAAATTGGAGTTGTTAGTCAAGATCCATTNNNNAGATCGGAAGAGCACACGTGACTCGCCAAGCTGAAGNNNNNNNNNNNNTCATTATGATACTAGAAAAGATGTTGAA |

**Supplemental Table 2.** Summary of repeated reactions and repools for MAD$^4$HatTeR and DR23K.

| Sample Type | Samples that underwent a second PCR/Capture n/N (%) | | Reactions/Captures that underwent repooling and resequencing n/N (%) | |
|---|---|---|---|---|
| | MAD$^4$HatTeR | DR23K | MAD$^4$HatTeR | DR23K* |
| Laboratory samples N = 28 | 5/28 (18%) | 28/28 (100%) | 5/33 (15%) | 35/56 (63%) |
| Field Samples N = 67 | 11/67 (16%) | 22/67 (33%) | 14/78 (18%) | 44/89 (49%) |

*Repooling was not performed for samples with <1 locus achieving >1 UMI coverage.

**Supplemental Table 3.** Number of genotype calls discordant between MAD$^4$HatTeR and DR23K by SNP.

| SNP | Number of discordant / Number of comparisons (%) | Prevalence of mutant allele (mixed or mutant) by MAD$^4$HatTeR | Prevalence of mutant allele (mixed or mutant) by DR23K |
|---|---|---|---|
| CRT C72S | 0 / 56 (0%) | 0 / 65 (0%) | 0 / 56 (0%) |
| CRT M74I / N75E / K76T | 1 / 56 (2%) | 1 / 65 (2%) | 0 / 56 (0%) |
| MDR1 N86Y/F | 0 / 54 (0%) | 0 / 64 (0%) | 0 / 55 (0%) |
| MDR1 Y184F | 13 / 59 (22%) | 51 / 65 (79%) | 41/59 (70%) |
| MDR1 S1034C | 0 / 63 (0%) | 0 / 65 (0%) | 0 / 63 (0%) |
| MDR1 N1042D | 0 / 63 (0%) | 0 / 65 (0%) | 0 / 63 (0%) |
| MDR1 D1246Y | 2 / 61 (3%) | 2 / 65 (3%) | 2 / 61 (3%) |
| DHFR A16V | 0 / 57 (0%) | 0 / 65 (0%) | 0 / 57 (0%) |
| DHFR N51I | 2 / 64 (3%) | 64 / 65 (99%) | 63 / 64 (98%) |
| DHFR C59R | 8 / 64 (13%) | 64 / 65 (99%) | 61 / 64 (95%) |
| DHFR S108N | 13 / 66 (20%) | 67 / 67 (100%) | 66 / 66 (100%) |
| DHFR I164L | 23 / 66 (35%) | 31 / 67 (46%) | 8 / 66 (12%) |
| DHPS I431V | 0 / 59 (0%) | 0 / 65 (0%) | 0 / 59 (0%) |
| DHPS S436A | 1 / 58 (2%) | 1 / 65 (2%) | 0 / 58 (0%) |
| DHPS S436F | 0 / 58 (0%) | 0 / 65 (0%) | 0 / 58 (0%) |
| DHPS A437G | 19 / 58 (33%) | 63 / 65 (97%) | 54 / 58 (93%) |
| DHPS K540E | 10 / 61 (16%) | 66 / 66 (100%) | 58 / 61 (95%) |
| DHPS A581G | 2 / 64 (3%) | 4 / 66 (6%) | 2 / 64 (3%) |
| DHPS A613S/T | 0 /63 (0%) | 0 / 65 (0%) | 0 / 63 (0%) |
| K13 P441A | 0 / 50 (0%) | 1 / 65 (2%) | 1 / 50 (2%) |
| K13 C469Y | 9 / 57 (16%) | 26 / 66 (40%) | 20 / 57 (35%) |
| K13 M476I | 0 / 62 (0%) | 0 / 66 (0%) | 0 / 62 (0%) |
| K13 Y493H | 0 / 56 (0%) | 0 / 66 (0%) | 0 / 56 (0%) |
| K13 R539T | 0 / 61 (0%) | 0 / 65 (0%) | 0 / 61 (0%) |

| | | | |
|---|---|---|---|
| K13 I543R | 0 / 61 (0%) | 0 / 65 (0%) | 0 / 61 (0%) |
| K13 R561H | 0 / 58 (0%) | 0 / 66 (0%) | 0 / 58 (0%) |
| K13 A578S | 2 / 59 (3%) | 4 / 66 (6%) | 3 / 59 (5%) |
| K13 C580Y | 0 / 63 (0%) | 0 / 66 (0%) | 0 / 63 (0%) |
| K13 A675V | 8 / 64 (13%) | 18 / 65 (28%) | 15 / 64 (23%) |

**Supplemental Figure 1**. Genomic regions of drug resistance genes targeted by DR23K and MAD$^4$HatTeR. The genomic region is indicated in gray, regions targeted by DR23K in green, and regions targeted by MAD$^4$HatTeR in blue. Known SNPs of interest are labeled in cyan.

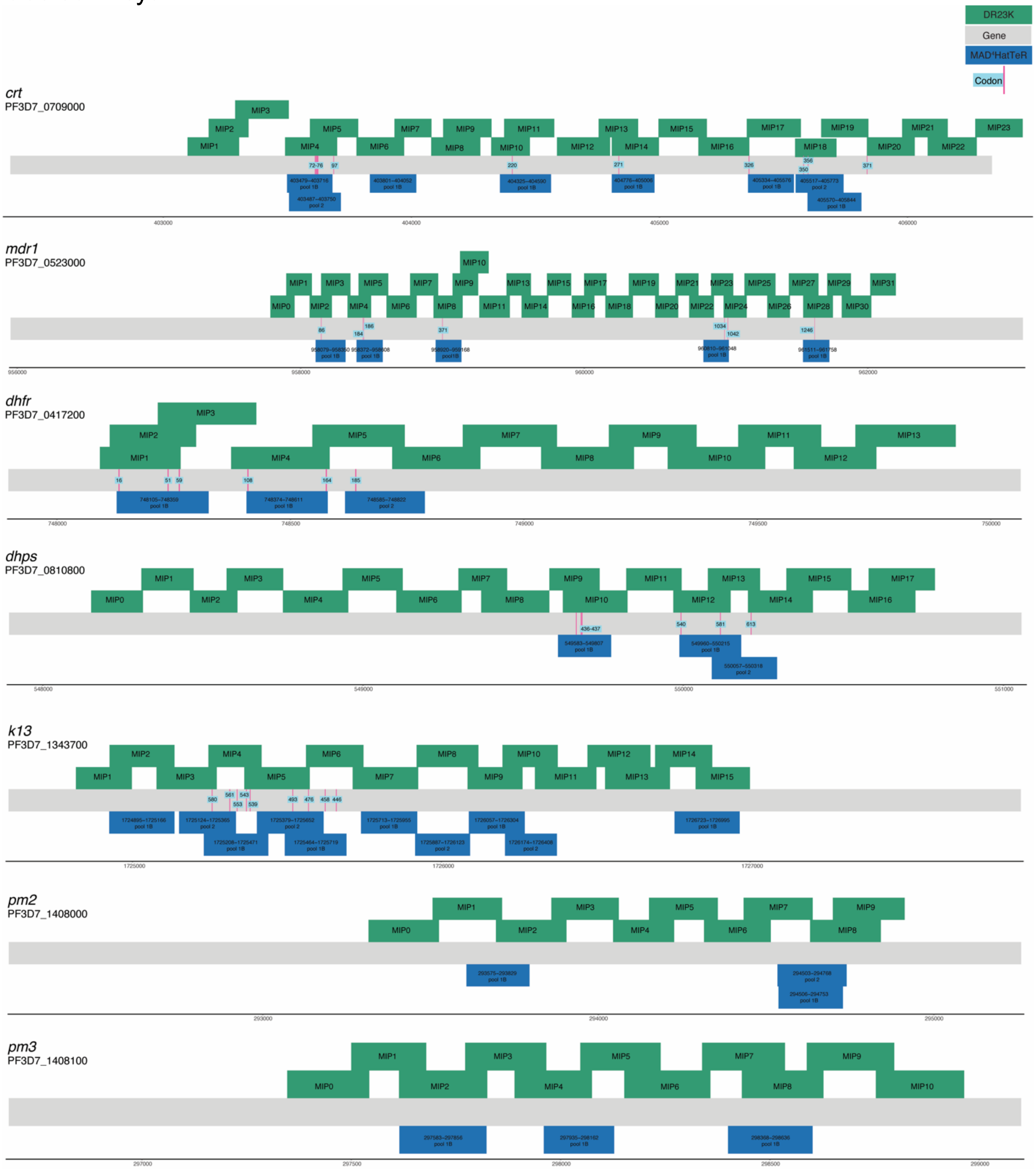

**Supplemental Figure 2**. Percentage of reads detecting variable drug resistance markers in DBS composed of mixed laboratory strains at varying proportions and parasite densities using MAD$^{4}$HatTeR. Absent bars indicate missing data (e.g. no genotypes were called). For the triple strain control, the expected percentages of the reference and alternative alleles are shown in the right panel for ease of comparison to the data on the left.

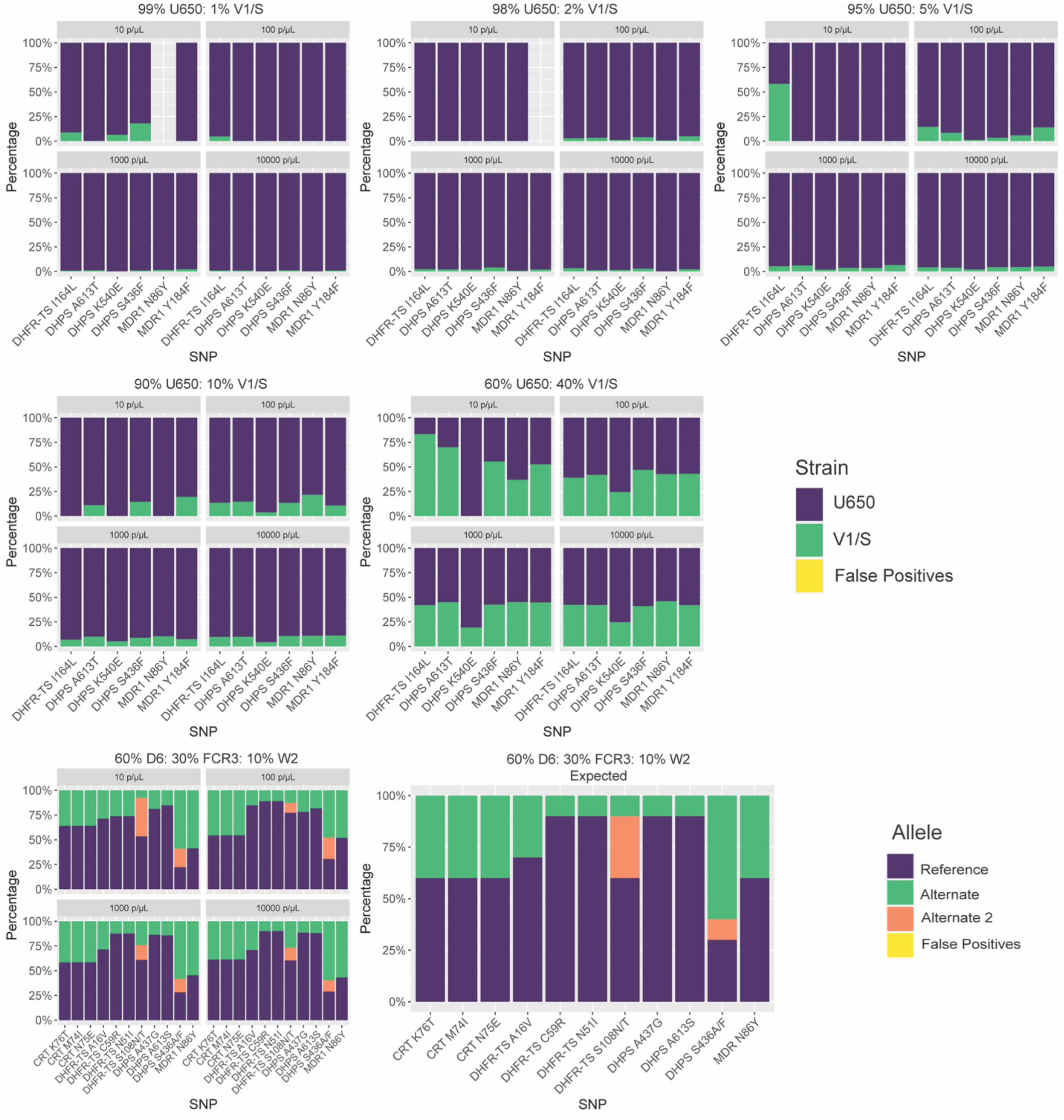

**Supplemental Figure 3.** Percentage of UMIs detecting variable drug resistance markers in DBS composed of mixed laboratory strains at varying proportions and parasite densities using DR23K. Absent bars indicate missing data (e.g. no genotypes were called). For the triple strain control, the expected percentages of the reference and alternative alleles are shown in the right panel for ease of comparison to the data on the left.

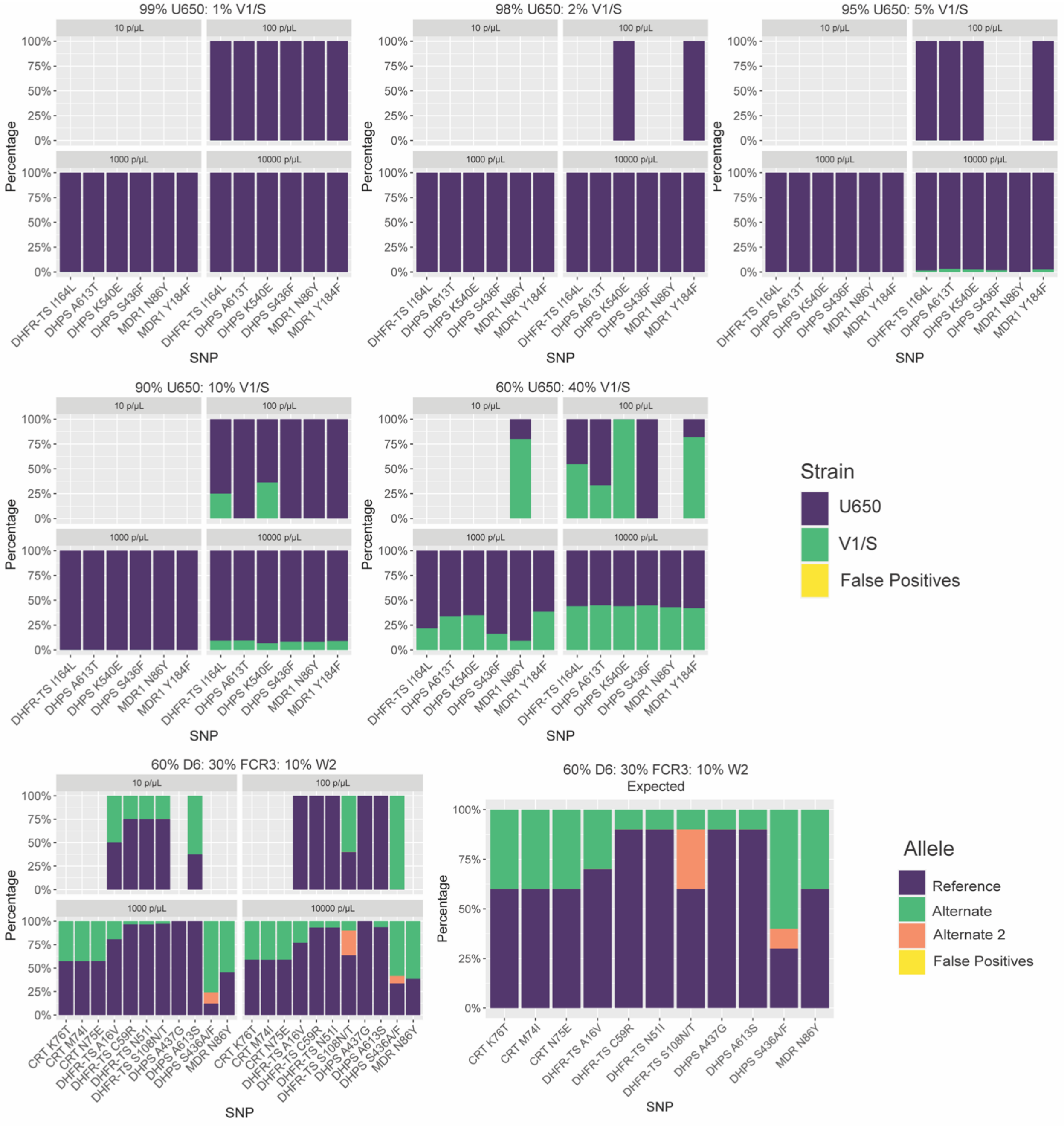

**Supplemental Figure 4.** Percentage of reads detecting variable microhaplotypes in DBS composed of mixed laboratory strains at varying proportions and parasite densities using MAD[4]HatTeR. Absent bars indicate missing data (e.g. no genotypes were called). For the triple strain control, the expected percentages of the reference and alternative alleles are shown in the right panel for ease of comparison to the data on the left.

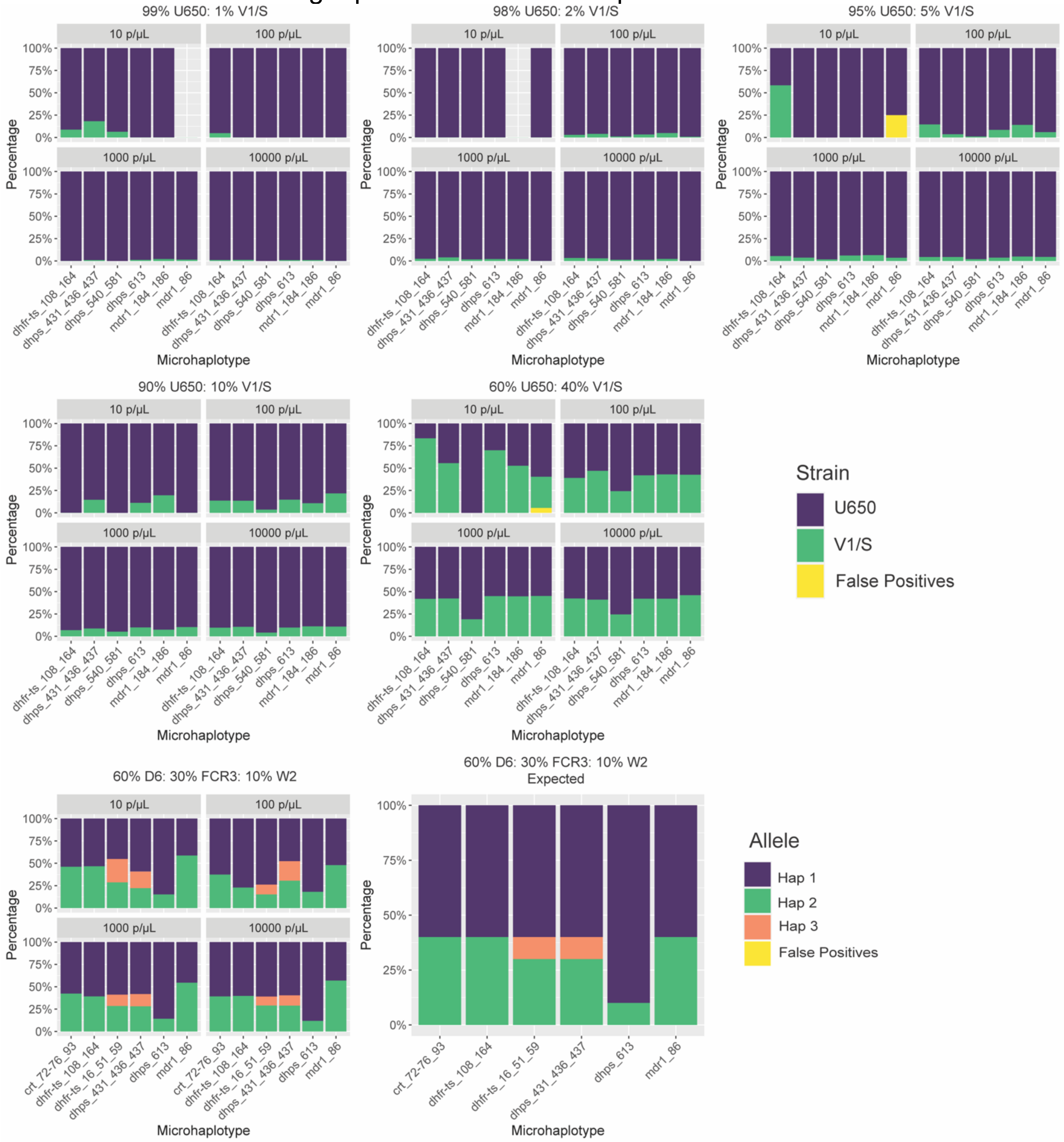

**Supplemental Figure 5.** Percentage of UMIs detecting variable microhaplotypes in DBS composed of mixed laboratory strains at varying proportions and parasite densities using DR23K. Absent bars indicate missing data (e.g. no genotypes were called). For the triple strain control, the expected percentages of the reference and alternative alleles are shown in the right panel for ease of comparison to the data on the left.

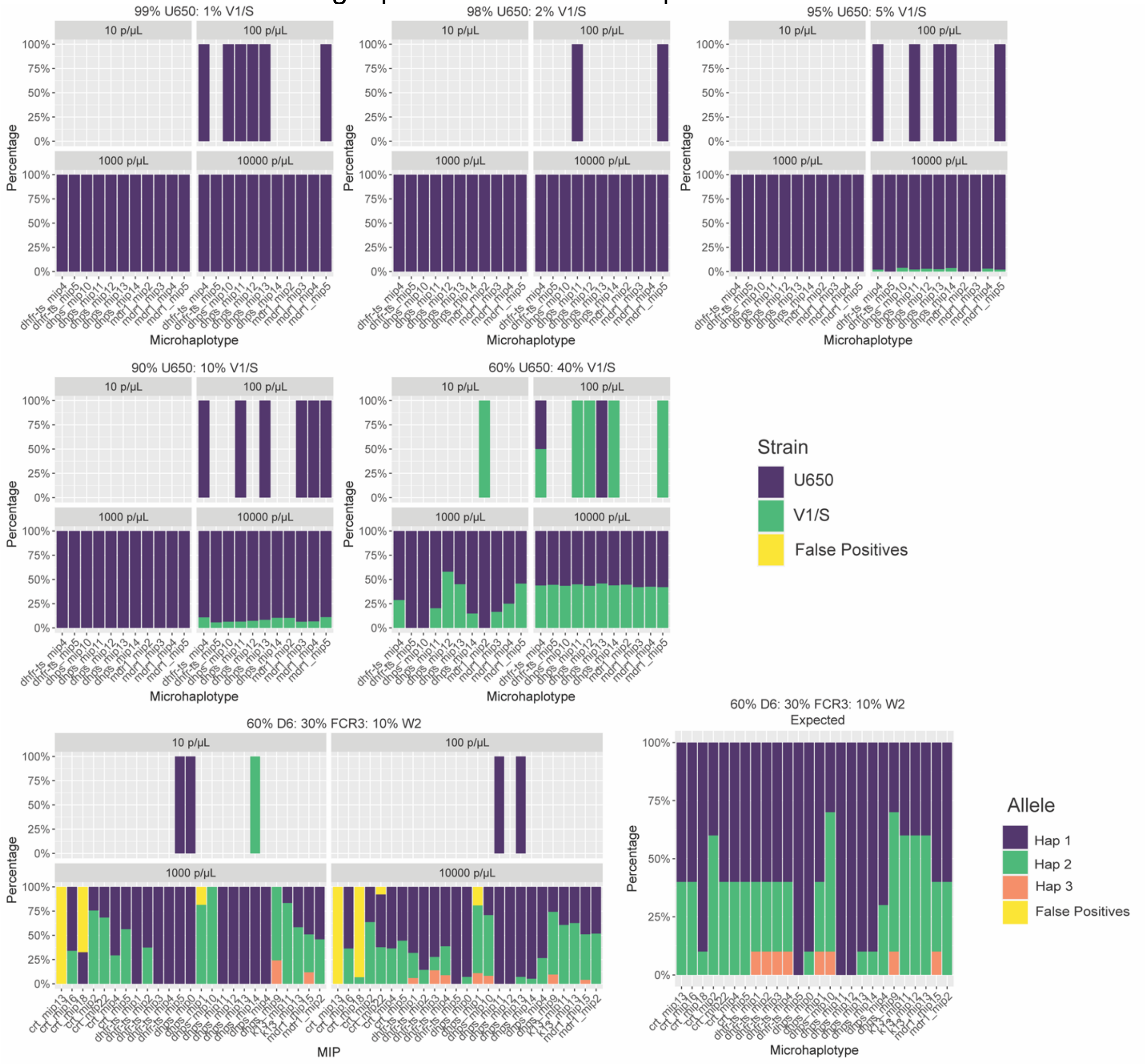

**Supplemental Figure 6.** Relationship between varATS qPCR-determined parasite densities and number of SNPs without successful genotype calls by DR23K. Each dot represents a field sample. Boxes show the first to third quartiles, and whiskers extend to the largest values no more than 1.5X the interquartile ranges. Statistical differences evaluated using pairwise Wilcox tests implemented in ggpubr:stat_compare_means function.

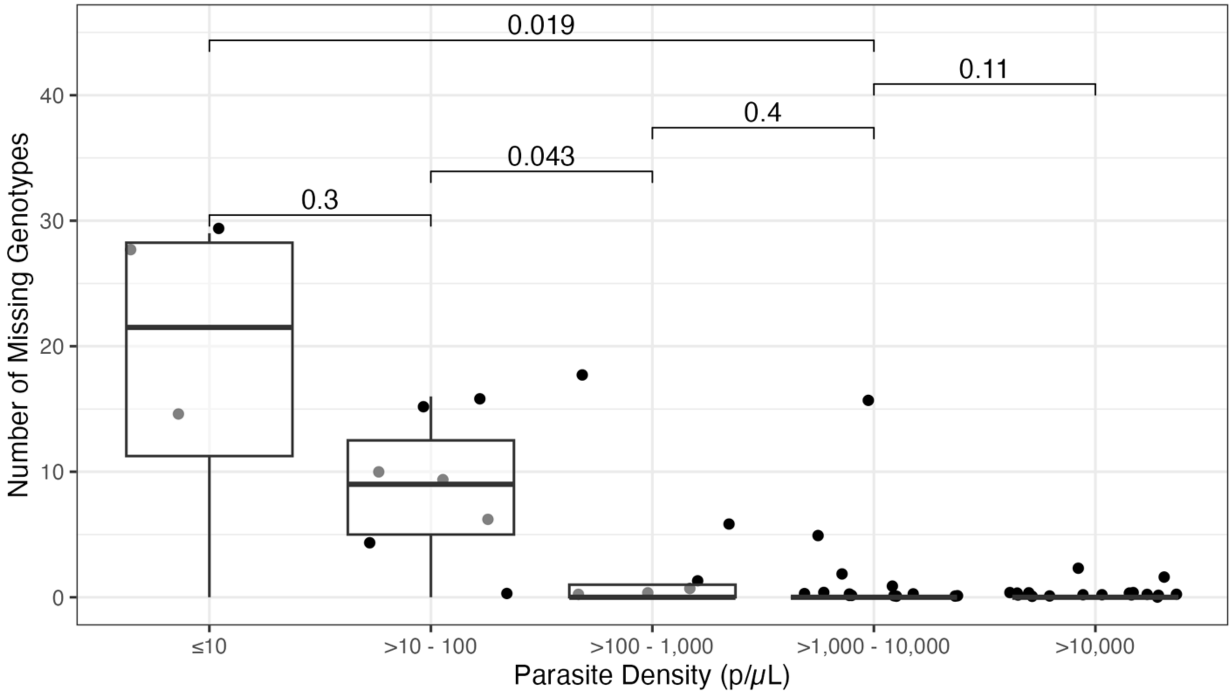

**Supplemental Figure 7.** The relationship between the number of genotype calls discordant between MAD[4]HatTeR and DR23K and varATS qPCR-determined parasite densities in field samples. A linear model of the log median parasite density as a function of the number of discordant SNP calls for a sample was fit to the data. A summary of the Pearson correlation and linear regression results are provided. The number of field samples contributing to the mean parasitemia for each category is indicated above the x-axis.

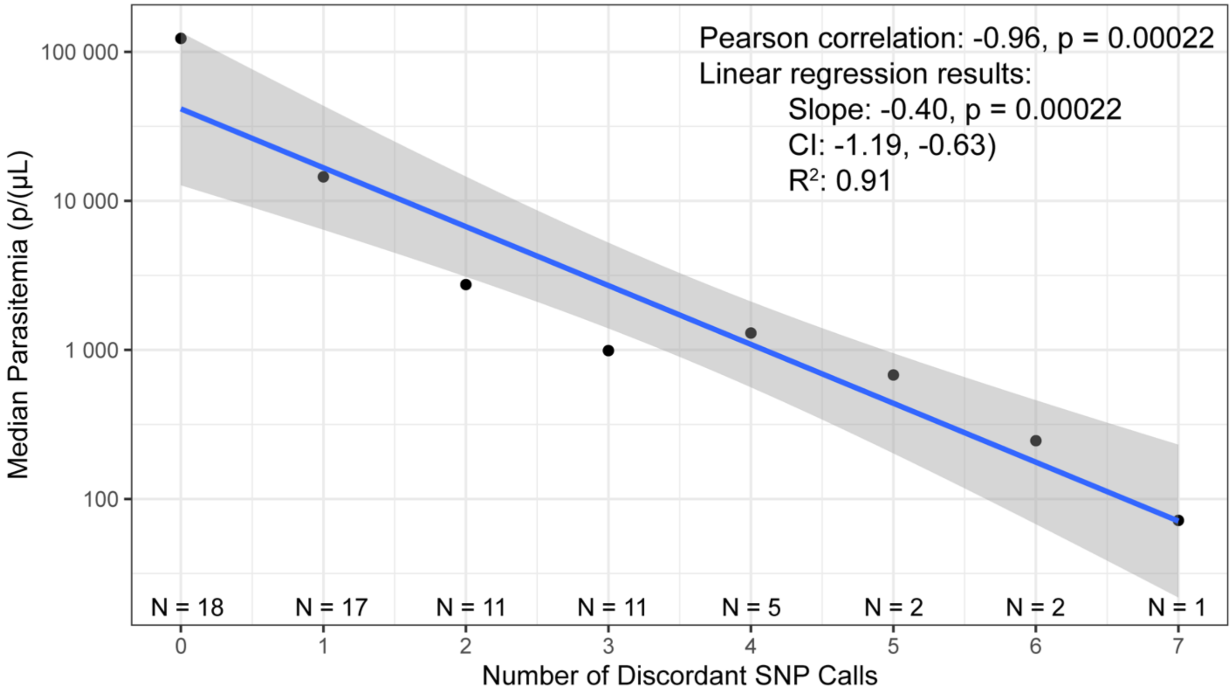

**Supplemental Figure 8.** Comparative differences between MAD$^4$HatTeR and MIP Assay Workflows. The schematic comparison illustrates the procedural differences between the MAD$^4$HatTeR and MIP assays, from initial reagent preparation through the final indexing PCR step. MAD$^4$HatTeR requires significantly more processing time, with approximately 6 hours needed to prepare a 96-well plate compared to 4 hours for the MIP assay. More importantly, the hands-on technologist time differs substantially: MAD$^4$HatTeR demands approximately 2 hours of active technical work, while the MIP assay requires only about 30 minutes. This operational efficiency enables laboratory staff to process multiple MIP plates simultaneously, a workflow advantage not feasible with the more labor-intensive MAD$^4$HatTeR procedure. Comparison of equipment and material requirements and post-PCR processing and quality control approaches can be found in Supplemental Methods.

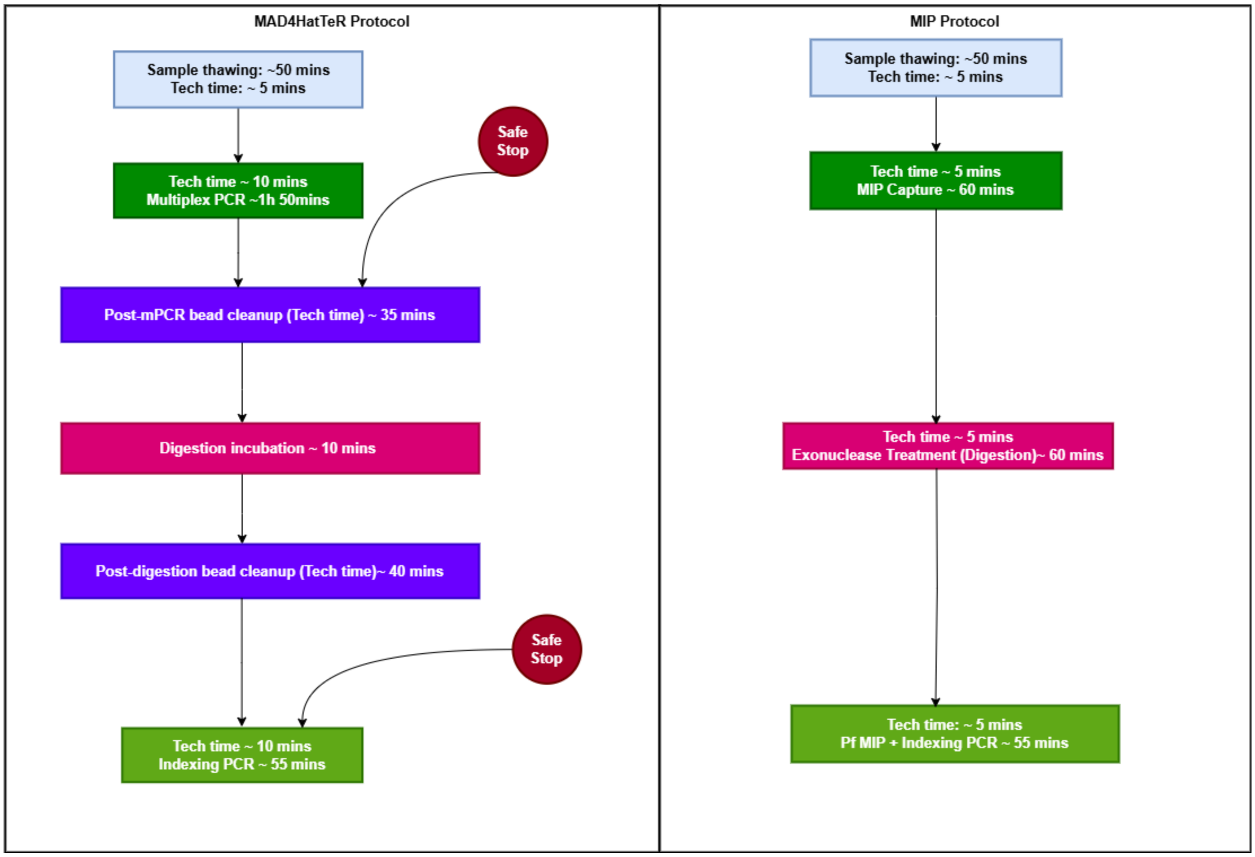

**Supplemental methods**

## MAD$^4$HatTeR sample pooling

The purpose is to combine samples to be run in Illumina sequencer as a pool.

A sample sheet was made and doublechecked to make sure that the sample names and indexes were not duplicated. The plates were briefly spun down and placed on the magnetic stand to separate beads. The samples were mixed into a single 1.5 mL microcentrifuge tube depending on the parasite concentrations as below.

- 30 µL for <10 parasites/µL
- 20 µL for 10 to <100 parasites/µL
- 15 µL for 100 to <1000 parasites/µL
- 10 µL for 1000 to <10000 parasites/µL
- 6 µL for 10000 to <100000 parasites/µL
- 3 µL for 100000 parasites/µL and above

After pooling, 1X volume of beads were added to the pool, vortexed and briefly spun down to collect liquids. The mixture was incubated for 5 minutes at room temperature, placed on a microcentrifuge tube magnetic stand, and incubated for 3 minutes or until the solution was clear. The supernatant was removed, the tube briefly spun down, placed back on the magnetic stand to remove the rest of the supernatant. 1.5 mL of 70% ethanol was added to the beads, and the tube rotated until the beads migrated to the opposite wall. After moving to the opposite wall, the ethanol supernatant was removed, the tube briefly spun down, placed back in the magnetic stand and the rest of the supernatant removed. The beads were left to dry at room temperature, until they looked matte and not shiny, but avoided cracking. The beads were resuspended with 43 µL TE buffer, incubated for 2 minutes and then put back on the magnetic stand for 3 minutes or until the liquid was clear. The TE was transferred to a clean, labeled tube ready for assessing the library purity with capillary electrophoresis.

## MIP capture, amplification, and sequencing

Oligonucleotides were synthesized as 200 nmole ultramers (Integrated DNA Technologies) with equimolar hand-mix option for random bases. The oligonucleotides were pooled as indicated in **Supplemental Table 1** to create the DR23K panel. Pools were 5’ phosphorylated using 1 µl (10 units) T4 polynucleotide 7 kinase (NEB) for every

nmole of probe, in 1X T4 DNA ligase buffer (NEB). Phosphorylation reactions were split into 50 µl aliquots, incubated in a thermocycler at 37°C for 45 min, followed by heat inactivation at 65°C for 20 min. Split reactions were pooled together for homogeneity, re-aliquoted, and kept at -20°C. Probes were diluted 1:8 in TE buffer (10 mM Tris, 1 mM EDTA, pH 8) to bring them to 1 µM working solution. Capture reactions were carried out as follows: 10 µl capture reactions for each sample containing Ampligase buffer (1X), Phusion DNA polymerase (0.0008 units/µl), Ampligase (0.04 units/µl), pooled MIPs (40 nM), dNTP (4 µM), and template DNA (5µl) were incubated in a preheated thermocycler at 95°C for10 min, 60°C for 1h, and then 4°C. Next, 2 µl of exonuclease mix containing 1X Ampligase buffer, 10 units exonuclease I, and 50 units exonuclease III were added to reactions with incubations at 37°C for 1 h, 95°C for 2 min, and then 4°C. The entire capture reaction (12 µl) was amplified in a 25 µl PCR reaction containing: 1X Phusion polymerase buffer, 1X Macromolecular Crowding (MMC) solution, 200 nM each dNTP, 0.02 units/µl Phusion DNA polymerase, and 500 nM each forward and reverse primer . PCR was performed using a preheated thermocycler at 98°C for 30 s, 22 cycles (98°C 10 s, 63°C 30 s, 68°C 30 s), 68°C for 2 min, and then 4°C. 50 ml 5X MMC was prepared by mixing the following components in water and filter sterilizing using a 0.2 µ nylon syringe filter: 3.75 g Ficoll 70 (GE Healthcare), 1.25 g Ficoll 400 (Sigma), and 0.125 g polyvinylpyrrolidone (Sigma). 5 µl of each capture was run on an agarose gel to assess reaction performance and inform sample pooling. Specifically, 5 µl of captures with a clear band on the gel and 10 µl of captures without a band were combined in a single tube, vortexed briefly, and centrifuged for 10 minutes. 90% of the supernatant was then transferred to a new tube, and cleaned using Ampure XP beads (Beckman Coulter) at 1.2x bead:DNA ratios using the manufacturer's protocol. Beads were eluted in 43 µl of TE buffer. 40 µl of the eluted sample was then run on a 1.5% agarose gel, and the band observed at ~500 bp was excised and underwent gel extraction using the Monarch DNA extraction kit (NEB). Libraries were then sequenced on an Illumina MIseq instrument using 150 bp paired-end sequencing with dual indexing using Mid-output Kit v2.

## Comparative differences between MAD$^4$HatTeR and MIP Assay Workflows Equipment and Material Requirements

Resource allocation differs markedly between the two methods. The MAD$^4$HatTeR protocol requires simultaneous use of two thermocyclers for the multiplex PCR step, effectively halving the potential throughput compared to the MIP assay, which requires only a single thermocycler per 96-well plate. This equipment requirement remains constant regardless of a laboratory's total thermocycler capacity, giving the MIP assay a considerable advantage in daily processing capacity.

Consumable usage represents another significant difference between the protocols. For each 96-well plate, MAD[4]HatTeR requires substantially more materials: 1248 P300/P200 barrier tips, 1,264 P10 tips, 2 PCR plates, and 12 plate sealants. By comparison, the MIP assay uses only 20 P300/P200 barrier tips, 288 P10 tips, 1 PCR plate, and 3 sealants—a substantial reduction in consumable requirements.

**Post-PCR Processing and Quality Control**

Following the indexing PCR step, amplicons from both methods can be stored at -20°C. This is crucial as it allows for batch processing of subsequent steps—including sample pooling, fragment size estimation, dimer detection, and library quantification—reducing overall turnaround time.

The protocols diverge in their quality control approaches after indexing. For MAD[4]HatTeR, the recommended practice is to evaluate approximately 6 representative samples and 2 positive controls from each plate using a Bioanalyzer or TapeStation to confirm successful library preparation. The MIP protocol, however, recommends spot checking of all samples to identify those with and without amplification bands, informing the pooling strategy. While the MIP protocol relies on this step for pooling decisions, MAD[4]HatTeR typically uses parasitemia data from either microscopy or varATS qPCR assays to guide pooling proportions.

Strategies to remove extraneous primers and probes also differ between methods. The MIP protocol routinely incorporates a gel clean-up step, whereas MAD[4]HatTeR only performs this step if dimer concentration exceeds 5% of the library pool. Before sequencing, both methods require library concentration estimation via Qubit quantification and fragment size analysis using either Bioanalyzer or TapeStation platforms. The entire post-PCR process—from pooling through quantification—takes approximately 8 hours for the MIP protocol compared to 4 hours for MAD[4]HatTeR when gel cleaning is not required.
